# Mechanisms of Intranasal Deferoxamine in Neurodegenerative and Neurovascular Disease

**DOI:** 10.3390/ph14020095

**Published:** 2021-01-27

**Authors:** Jacob Kosyakovsky, Jared M. Fine, William H. Frey, Leah R. Hanson

**Affiliations:** 1 School of Medicine, University of Virginia, 200 Jeanette Lancaster Way, Charlottesville, VA 22903, USA; jk6bw@hscmail.mcc.virginia.edu; 2 HealthPartners Neuroscience Center, HealthPartners Institute, Saint Paul, MN 55130, USA; alzheimr@umn.edu (W.H.F.II); Leah.R.Hanson@healthpartners.com (L.R.H.)

**Keywords:** intranasal, deferoxamine, Alzheimer’s disease, Parkinson’s disease, ischemic stroke, intracranial hemorrhage, iron chelation, brain iron dyshomeostasis

## Abstract

Identifying disease-modifying therapies for neurological diseases remains one of the greatest gaps in modern medicine. Herein, we present the rationale for intranasal (IN) delivery of deferoxamine (DFO), a high-affinity iron chelator, as a treatment for neurodegenerative and neurovascular disease with a focus on its novel mechanisms. Brain iron dyshomeostasis with iron accumulation is a known feature of brain aging and is implicated in the pathogenesis of a number of neurological diseases. A substantial body of preclinical evidence and early clinical data has demonstrated that IN DFO and other iron chelators have strong disease-modifying impacts in Alzheimer’s disease (AD), Parkinson’s disease (PD), ischemic stroke, and intracranial hemorrhage (ICH). Acting by the disease-nonspecific pathway of iron chelation, DFO targets each of these complex diseases via multifactorial mechanisms. Accumulating lines of evidence suggest further mechanisms by which IN DFO may also be beneficial in cognitive aging, multiple sclerosis, traumatic brain injury, other neurodegenerative diseases, and vascular dementia. Considering its known safety profile, targeted delivery method, robust preclinical efficacy, multiple mechanisms, and potential applicability across many neurological diseases, the case for further development of IN DFO is considerable.

## 1. Introduction

As the leading cause of global disability and 2nd leading cause of death, the burden of neurological disease is tremendous [1]. Treatment options remain disappointingly limited for the majority of these conditions, especially aging-related diseases such as Alzheimer’s disease (AD) [2,3], other dementias [4], and stroke [5]. Many challenges have contributed to this therapeutic gap including the need to deliver therapeutics in meaningful concentrations across the blood-brain barrier (BBB) [6], elusive disease etiologies [7,8,9], and complex disease mechanisms [3,8,10,11,12], among others. Although the past decade has yielded incredible insight into disease pathogenesis, these discoveries have largely failed to translate into clinical benefit [13,14,15,16]. It is of paramount importance to fill this therapeutic gap in our aging population.

In this review, we present the evidence for intranasal (IN) delivery of deferoxamine (DFO), a metal chelator that has shown promise in both preclinical and early clinical studies in AD, Parkinson’s disease (PD), ischemic stroke, and intracranial hemorrhage. Iron chelation counters several disease-specific processes via disease-nonspecific mechanisms, and brain iron accumulation represents an untapped and highly accessible therapeutic target across many neurological diseases.

## 2. The Development of Deferoxamine and Intranasal Delivery

DFO, developed over half a century ago, is the most potent and widely used of several FDA-approved iron chelators [17,18]. These therapeutics were originally developed to address systemic iron overload states such as transfusion-dependent thalassemia major but have since seen significant preclinical and clinical development for use across cancer [19,20,21], imaging [22,23], and neurological disease [24,25,26,27,28,29,30,31,32,33,34,35,36,37,38,39,40,41,42]. DFO has a short plasma half-life and is typically challenging to deliver systemically, with most delivery methods involving long courses of intravenous (IV) infusion or intramuscular (IM) injection [43]. Such routes struggle to achieve meaningful therapeutic concentrations of drug in the brain due to the blood-brain barrier (BBB) [24], a common problem in neuropharmacology [6]. Furthermore, at the higher doses required to achieve such concentrations, DFO can cause systemic toxicity [44].

In 1989, William H. Frey II discovered the non-invasive intranasal method of bypassing the BBB to deliver and target neurological therapeutic agents to the brain and filed the first patent [45,46]. The IN route bypasses the BBB by rapidly delivering therapeutics extracellularly along the olfactory and trigeminal nerve pathways to the CNS [24,47,48,49,50,51,52]. Over the past three decades, IN delivery has been shown to provide a safe and efficacious means to overcome these challenges and has remarkably transformed the problem of delivering therapeutics to the central nervous system (CNS) [47,48]. IN delivery has been successfully and robustly applied to a wide array of pharmacologic agents, from small molecules to peptides [50], oligonucleotides [53], stem cells, and immune cells [54,55,56,57]. IN delivery minimizes systemic drug exposure while resulting in comparable or higher CNS concentrations compared to alternate methods of delivery [24,47,48,58]. IN delivery is increasingly considered the future of CNS pharmacotherapy, and its emergence is perhaps best attested by the use and efficacy of IN insulin in several clinical trials for AD [59,60,61,62].

The IN delivery of DFO achieves micromolar concentrations in the brain within minutes and offers up to 200-fold greater targeting compared to IV delivery in rodents [24,58]. In a recent review, Farr and Xiong discuss the formulation and delivery of DFO in detail, tabulating studies performed to date across AD, PD, and intracerebral hemorrhage [28]. In this review, we cast the spotlight on IN DFO and its therapeutic mechanisms for human neurological disease. In addition to evidence of IN DFO as a treatment for AD [63], PD [64], and stroke [65], decades of work across a number of research groups investigating systemically administered DFO and other iron chelators support this treatment approach.

## 3. Iron, Chelation, and the Brain

Iron is essential to a number of physiologic processes within the brain including oxygen transport, neurotransmission, and myelin homeostasis. Elaborate mechanisms transport, distribute, and store iron for use by intracellular iron-binding proteins [66]. However, a fraction of cellular iron that is not protein-bound, labile iron, is highly pro-oxidant. This intracellular labile iron pool is capable of catalyzing the production of hydroxyl radicals, contributing to oxidative stress and cellular damage [42,67,68,69]. Furthermore, labile iron catalyzes the cascade of lipid peroxidation that drives ferroptosis, a recently discovered mechanism of degenerative cell death [68,69]. Brain iron homeostasis is a delicate balance of the need for iron and its toxicity.

In its simplest mechanism, iron chelation therapy sequesters and clears the labile iron pool, thus counteracting oxidative damage resulting from pathologic iron accumulation [18,43,67,70]. As discussed in further detail below and elsewhere, this mechanism has direct relevance to a number of neurological disorders including AD, PD, progressive supranuclear palsy (PSP) [71], ischemic stroke, and intracranial hemorrhage, where iron dyshomeostasis—disruption of the iron balance leading to iron accumulation and consequent toxicity—is a known potential contributor to disease pathogenesis and neuronal injury [12,41,42,68,72,73,74]. Although current evidence strongly suggests that iron overload is associated with oxidative stress and injury in these states, it is unlikely that reversal of iron accumulation alone will reverse the underlying disease processes.

**Figure 1:**
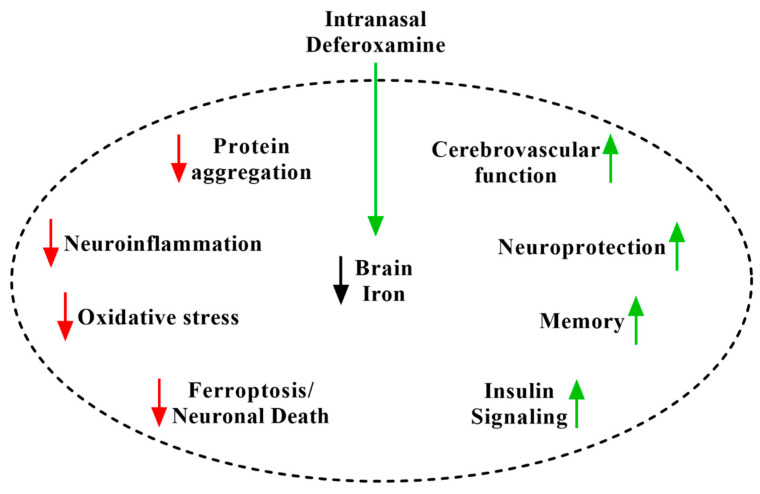
The disease-nonspecific mechanisms of iron chelation using intranasal deferoxamine (IN DFO). Depletion of brain iron engages a number of disease-relevant pathways.

Instead, we suggest that the major mechanism of iron chelation treatment of neurological disease is the disease-nonspecific depletion of brain iron causing engagement of several biochemical pathways that intersect with and potentially slow disease-specific pathogenesis. The depletion of iron activates hypoxia-inducible factor 1α (HIF-1α) by destabilizing its regulatory prolyl-hydroxylase, an iron-dependent protein [75,76]. HIF-1α activates an elaborate transcriptional program designed as an organized cellular response to hypoxia, mediated principally by vascular endothelial growth factor (VEGF), erythropoietin (EPO), inducible nitric oxide synthase (iNOS), and insulin-like growth factor (IGF) [40,75,76]. By these pathways, iron chelators including IN DFO have been found to reverse the accumulation of protein aggregates [28,30,33,34,77,78,79], suppress neuroinflammation [31,38,77,80,81,82,83,84], protect against oxidative stress and neuronal injury [30,31,32,36,81,83,85], improve cerebrovascular function [25,37,40,76], activate pro-survival signaling pathways [30,31,32,33,34,35,36,76], bolster cerebral glucose metabolism [30,31,35], and strengthen synaptic function [35,86,87] (Figure 1). In the following paragraphs, citing the considerable body of preclinical and early clinical research for IN DFO and other iron chelators, we argue multipronged mechanisms by which engagement of these pathways helps to counter pathogenesis across neurological disease.

## 4. IN DFO for Alzheimer’s Disease

Alzheimer’s disease (AD) is the most common dementia, afflicting a staggering 50 million individuals worldwide [2]. A seminal two-year single-blind clinical trial found that intramuscular administration of DFO significantly reduced the rate of decline in living skills for AD patients by approximately twofold [39]. In animal models of AD, evidence for the therapeutic efficacy of DFO, IN DFO, and other iron chelators is very strong and has been reviewed extensively elsewhere [28,88]. From the work of multiple research groups, IN DFO reduces cognitive decline and pathological hallmarks in the APP/PS1, P301L, and ICV STZ rodent models of AD [30,31,33,34,35,36,77,89]. Likewise, the preclinical evidence for other iron and metal chelators is also strong across these models [42,70,72].

**Figure 2:**
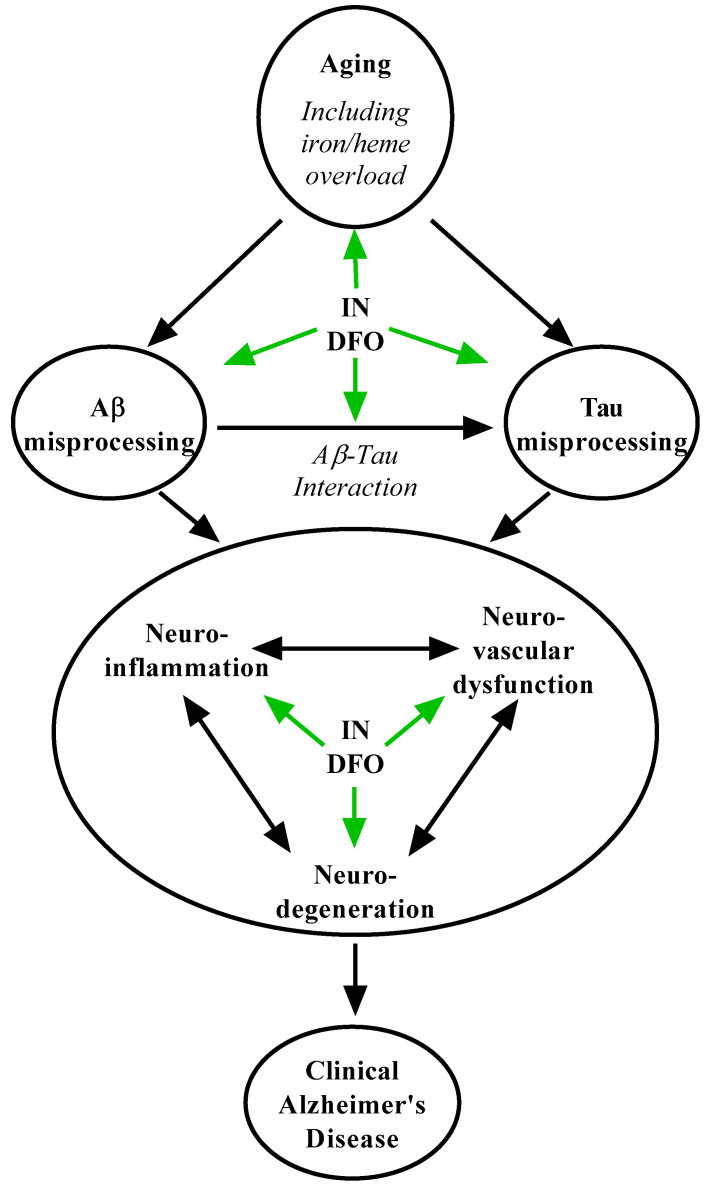
Putative mechanisms of IN DFO in Alzheimer’s disease. IN DFO engages Alzheimer’s pathogenesis at multiple levels, including a number of contributory aging-related processes, amyloid misprocessing, tau misprocessing, amyloid-tau interaction, neuroinflammation, neurovascular dysfunction, and neurodegeneration.

For many years, the amyloid hypothesis has dominated research on and understanding of AD pathoetiology [90], but the repeated failure of therapeutics targeting amyloid-β (Aβ) has undermined testimony of its accuracy [7,91]. Rather, converging lines of evidence suggest an alternative paradigm defining AD as a spectrum of decline caused by the interaction of aging-driven Aβ and tau neuropathology (Figure 2). In this framework, aging drives abnormal tau processing driving neurodegeneration [92,93,94] with Aβ pathology accelerating [91,93,94,95] and contributing to [90,91,95,96] this process, reconciling spatiotemporal pathological data across the aging population [8,94,97] with the causes of familial forms of AD [90]. The process of consequent cognitive decline appears to involve a dynamic interplay of progressive synaptic dysfunction, neurodegeneration [69,91,95], neuroinflammation [92,98], neurovascular breakdown [99], and the collapse of large-scale brain networks [100]. IN DFO can act on multiple processes in this cascade to slow disease progression. DFO has been shown to reduce Aβ aggregation [31,33,35,77] and hyperphosphorylation of tau [34,101], and involvement of transition metals including iron is thought to contribute to the toxicity of these pathological hallmarks [70,73]. DFO is a robust inhibitor of glycogen synthase kinase-3β (GSK-3β) [30,31,32,34], a well-established therapeutic target in tauopathy, and DFO was found to reverse tau-mediated neurodegeneration in rabbits treated with aluminum [101]. GSK-3β is also hypothesized as a major link between accumulation of Aβ and consequent hyperphosphorylation of tau in AD [91,93,96], placing DFO in position to suppress this acceleratory process. Additionally, iron accumulation and iron-mediated neuronal ferroptosis have been increasingly implicated as contributing to AD neurodegeneration [69,72,73], and IN DFO directly suppresses this process including iron-associated oxidative stress [42,78]. DFO likewise ameliorates neuroinflammation [30,38,81,82,83] which is hypothesized to contribute to Aβ toxicity and damage in AD.

Acting by its disease-nonspecific mechanism of HIF-1α activation, DFO appears to operate at a number of further levels countering the AD process. In two clinical trials, systemic administration of DFO strongly improved cerebral vasoreactivity and autoregulation especially in older individuals [37,40]. Decline in neurovascular function is thought to contribute to and be a part of AD pathogenesis [99]. DFO also activates glucose transporters including glucose transporter 1 (GLUT1) via HIF-1α [35,75,76], countering cerebral hypometabolism that is an early hallmark of AD. Furthermore, DFO has been robustly associated with activation of the insulin signaling pathway [30,31,32,34,38]. Activation of this pathway is known to act on astrocytes, microglia, and neurons to suppress neuroinflammation and promote neuroplasticity [102], and indeed, intranasal administration of insulin has shown benefit in several Phase 2 AD trials [60,61,62]. Finally, there is also precedent for DFO to improve memory in the absence of disease with IN DFO improving memory in healthy mice [32], possibly via GSK-3β mediated neuroplasticity [102].

Additionally, accumulating free iron may inactivate the human brain muscarinic cholinergic receptor (mAChR), contributing to impaired memory in AD [103]. Further, free heme, which contains iron, increases in the brains of AD patients, and like free iron inactivates the mAChR. A 2.5-fold increase in heme was also reported in the temporal lobe of deceased individuals with AD [104]. Consistent with this finding, the level of ferrochelatase in AD temporal lobe was 4.2 times that in nondemented controls, suggesting up-regulated heme synthesis [104]. In vitro, DFO protects the mAChR from inactivation by both iron and heme [Frey WH 2nd, Bordayo EZ and Hanson LR unpublished results]. Relatively little attention has been paid to the fact that Aβ binds both iron and heme. Heme binding to Aβ prevents Aβ aggregation by forming an Aβ–heme complex, and this Aβ–heme complex has peroxidase activity [105]. Phylogenic variations in the amino acid sequence of Aβ explain tight heme-binding to human Aβ and have been proposed to contribute to the increased human susceptibility to AD [106].

Thus, the positive early systemic clinical trial [39], the overwhelming body of preclinical evidence, and multimodal mechanisms of action strengthen the argument for further development of IN DFO for AD.

## 5. IN DFO for Parkinson’s Disease

Parkinson’s disease (PD), one of the most common movement disorders, continues to challenge those developing disease-modifying therapies. DFO has been shown to be strongly neuroprotective in several induced rodent models of PD [28], reversing motor deficits and improving survival of dopaminergic neurons following administration of 6-hydroxydopmaine (6-OHDA) [107,108,109], rotenone, methyl-phenyl-tetrahydropyridine (MPTP) [110], and α-synuclein [29]. Other iron chelators have extensively paralleled these findings [70,108,111]. Notably, the iron chelator deferiprone has shown promise in early clinical trials [111], although systemic administration and lower affinity for iron potentially limit this therapy’s efficacy compared to IN DFO.

**Figure 3:**
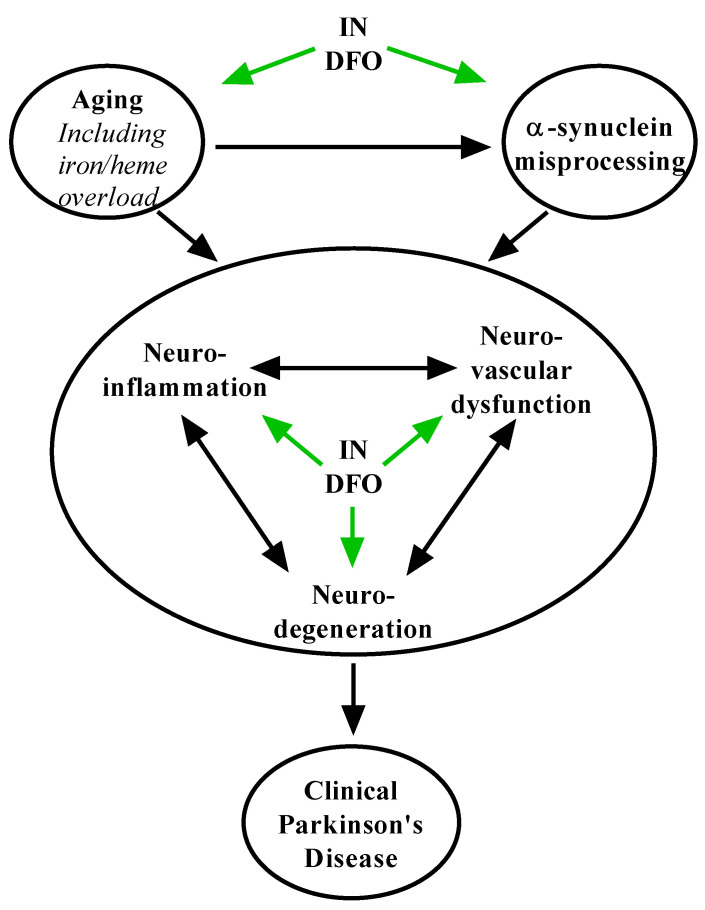
Putative mechanisms of IN DFO in Parkinson’s disease. IN DFO engages pathways that limit age-related processes such as iron accumulation, α-synuclein misprocessing, neuroinflammation, neurovascular dysfunction, and neurodegeneration to counter the Parkinson’s pathological process and loss of dopaminergic neurons.

DFO may act by multiple mechanisms to slow the progression of PD, many overlapping with its role in AD (Figure 3). Although complete detail remains elusive, PD is thought to be driven by the age-related pathological accumulation of α-synuclein aggregates into Lewy bodies [10]. This process results in progressive oxidative stress, lysosomal and mitochondrial dysfunction, and neurodegeneration, with possible roles for prion-like pathological spread and autoimmunity. Dopaminergic neurons within the substantia nigra pars compacta (SNpc) are particularly susceptible to this process [10,13], causing motor deficits in PD. DFO has been shown to directly reduce the expression and aggregation of α-synuclein [29,110]. Furthermore, it is well established that iron selectively accumulates within the SNpc and is associated with its exquisite vulnerability to oxidative stress and ferroptosis [42,69,72,73], and administration of DFO reverses this process [72,78]. DFO administration activates insulin signaling and glucose metabolism [32,34,89,107], and other modulators of these processes, including IN insulin, have similarly shown substantial preclinical efficacy in PD [112]. Finally, via its activation of the HIF-1 response and neurotrophic growth factors [35,86,87], DFO strongly promotes neuronal survival, mechanisms which may in themselves check the progression of degenerative processes in the PD brain. Thus, there is overwhelming evidence and rationale for further development of IN DFO and other iron chelators in PD.

## 6. IN DFO for Ischemic Stroke

Ischemic stroke is one of the leading causes of mortality and disability. Therapeutic use of DFO has shown considerable preclinical efficacy in animal models of stroke. IN DFO was found to significantly reduce infarct volume with middle cerebral artery occlusion (MCAO) in rats with either pre- or post-treatment [24]. These results are supported by efficacy of systemically administered DFO in the same model [113], as well as in rat ischemia induced by carotid ligation [114]. Furthermore, systemic DFO has been shown to cause robust ischemic preconditioning in the rodent brain, a strategy which is thought to ameliorate further damage due to multiple occlusive events, a common complication of stroke [76,115,116,117]. These results are similarly supported by a considerable body of similarly positive results from other iron chelators [70], supporting a real therapeutic role for this strategy in stroke treatment.

**Figure 4:**
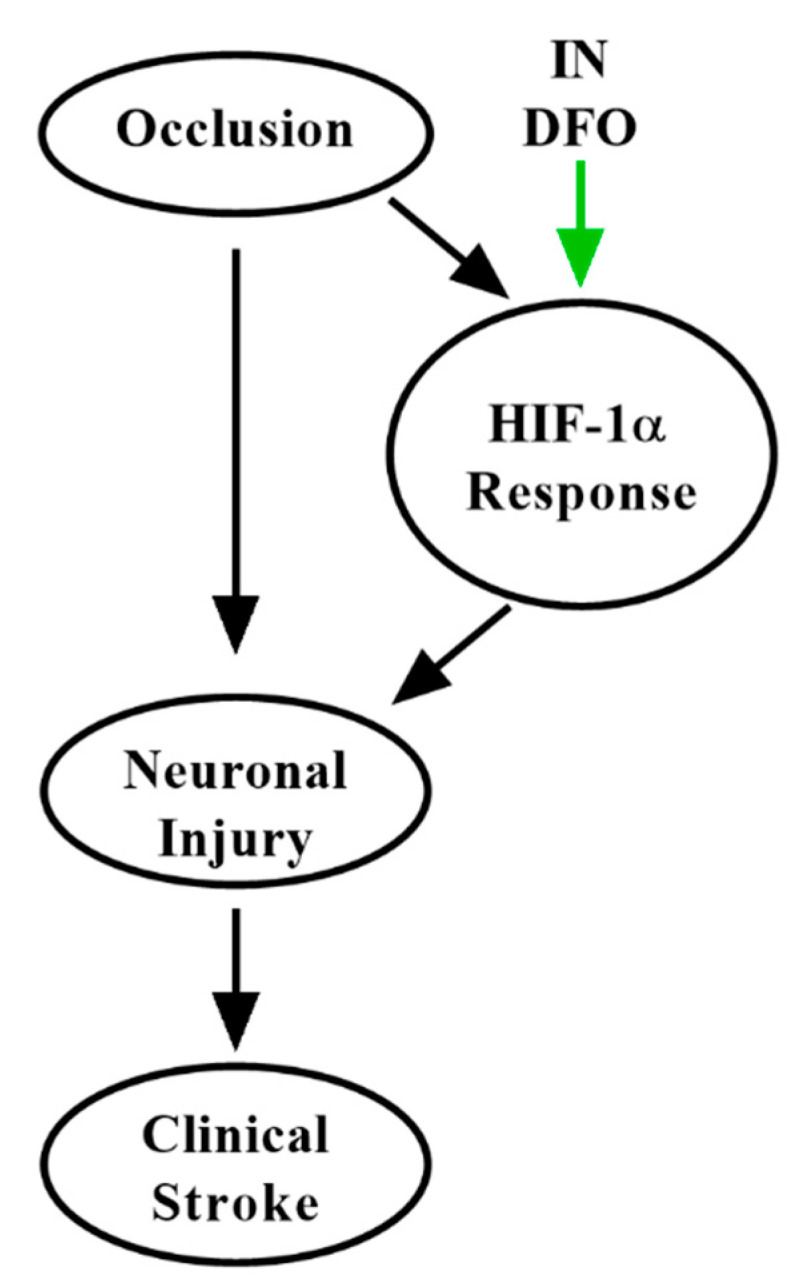
Putative mechanisms of IN DFO in ischemic stroke. IN DFO bolsters the brain’s intrinsic hypoxic response, contributing to immediate neuroprotection, improved cerebrovascular function, and ischemic preconditioning to minimize the impact of future occlusive events.

Occlusive stroke causes a complex pathophysiologic cascade of ischemic injury. Neurons are exquisitely sensitive to hypoxia, which causes cell death by a number of intersecting mechanisms [11]. By depleting cellular iron, DFO acts as a hypoxia-mimetic [115], bolstering the HIF-1α pathway and thus multiple aspects of the brain’s inherent neuroprotective defense against ischemia [37,40,75]. Additionally, by preconditioning the hypoxic response, DFO may ameliorate damage due to further ischemic events [113,116]. It is increasingly established that cerebral edema and hemorrhagic conversion also participate in damage following ischemic stroke [11], and DFO strongly reduces toxicity associated with these processes due to chelation of redox-active iron. Thus, as in AD and PD, DFO holds considerable promise to modify multiple aspects of the pathogenesis of ischemic stroke (Figure 4) to rescue functional impairment, a feature lacking in many putative strategies developed to date [41]. The speed, CNS targeting, and safety of IN delivery further strengthens this approach.

## 7. IN DFO for Intracranial Hemorrhage

**Figure 5:**
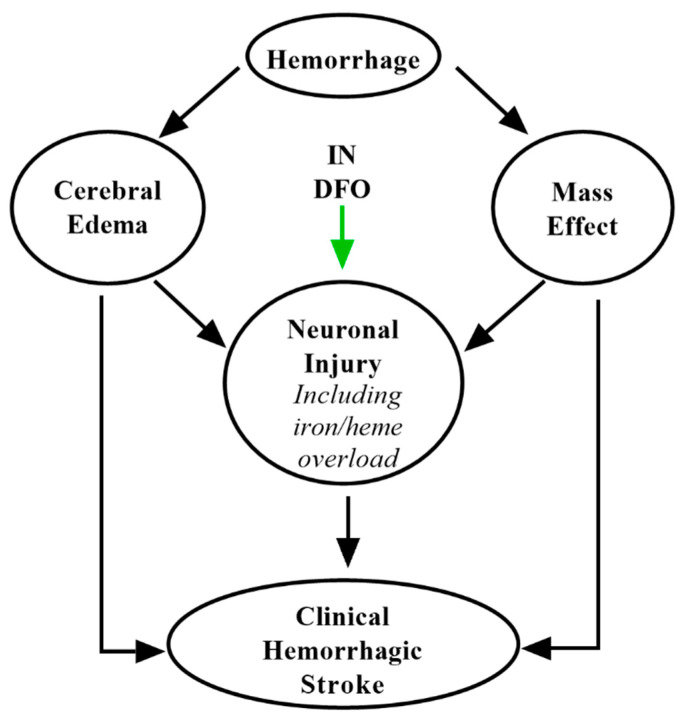
Putative mechanisms of IN DFO in intracranial hemorrhage. IN DFO minimizes the impact of heme- and iron-mediated neurotoxicity following hematoma.

DFO has been perhaps best studied in the context of intracranial hemorrhage (ICH), hemorrhagic events constituting around 15% of all strokes. Its substantial preclinical evidence in intracerebral hemorrhage and subarachnoid hemorrhage as well as mechanisms have been extensively reviewed elsewhere [27,85,118]. Succinctly, DFO is thought to chelate redox-active iron released from hemoglobin and myoglobin following ICH [12], minimizing secondary brain damage (Figure 5). Notably, the i-DEF trial evaluating IV DFO after intracerebral hemorrhage did not suggest therapeutic benefit [118]. However, this study was limited by systemic toxicity associated with higher doses of IV DFO, and the low doses studied may not have been sufficient to generate adequate CNS coverage following ICH [119]. IN administration may overcome this hurdle by targeting DFO to the CNS (up to 200-fold) as compared to systemic modes of delivery [24]. Another trial found that administration of DFO positively impacted several endpoints following ICH, although the investigators concluded more conclusive trials were required [120]. Given its substantial preclinical efficacy and the advantages of IN delivery, further investigation of IN DFO in ICH is still warranted.

## 8. IN DFO for Other Neurological Diseases

The wide-ranging efficacy of IN DFO in neurologic disease largely stems from its disease-nonspecific mechanism intersecting disease processes at multiple pathogenic nodes. Targeting multiple pathologies is an advantage for the treatment complex disorders such as in AD and expands the applicability of IN DFO to other neurological disorders. For example, DFO shows promise as an agent to improve memory in aging and other dementias. DFO was shown to reduce cognitive decline with aging [121], and IN DFO improves baseline performance on a memory task in young, healthy mice [32]. In these settings, it is possible that DFO may simply promote neuronal functioning through chelation of age-associated redox-active iron. Alternatively, its mechanisms promoting synaptic function, glucose metabolism, or cerebrovascular function may play a role and merit further investigation. In multiple sclerosis, brain iron is found to accumulate independently of inflammation and is increasingly thought to contribute to pathogenesis [74]. DFO was found to suppress inflammatory damage in animal models [80,122] and was tolerated in early clinical studies of this disease [123,124]. Insomnia-associated cognitive decline is associated iron accumulation, oxidative stress, and inflammation [73], and may likewise be ameliorated with IN DFO treatment. Furthermore, accumulation of iron is thought to be one of the many pathophysiological sequelae of traumatic brain injury (TBI) [125]. DFO has been shown to suppress this post-TBI iron overload as well as reverse hydrocephalus and cognitive deficits following TBI in animal models [79,126,127,128]. The sequelae of TBI combine neurodegenerative, neuroinflammatory, and both ischemic and hemorrhagic neurovascular components [125], a perfect model wherein DFO may uniquely enact beneficial impact through its multiple intersecting disease-modifying mechanisms.

Finally, there is evidence for the development of IN DFO in vascular dementia, the second leading cause of dementia worldwide. DFO has been shown to reverse cognitive deficits following hemorrhagic and ischemic vascular events in animal models, including events induced by diabetes [25]. Vascular dementia is a highly heterogenous entity involving generally stepwise cognitive decline secondary to progressive ischemic brain injury, and is at the crossroads of neurovascular dysfunction, neurodegeneration, and metabolic syndrome [129]. As in TBI, DFO may uniquely operate at the center of this pathological process modifying each of its components. DFO may prevent cognitive dysfunction secondary to ischemia via activation of the HIF-1α response, providing neuroprotection following occlusive events akin to its role following ischemic stroke [24,76]. Furthermore, DFO may provide ischemic preconditioning within the brain, preventing further decline caused by progressive neurovascular dysfunction [86,113,115,116,117]. Moreover, by improving cerebrovascular function, DFO may prevent much of this neurovascular decline with aging [37,40]. Additionally, neurodegenerative changes are often associated with vascular dementia [129], and thus DFO’s pro-survival mechanisms in PD and AD are relevant to progression in this condition. Lastly, metabolic syndrome and brain insulin resistance have been increasingly associated with the spectrum of neurodegeneration and neurovascular decline [102,129]. DFO activates insulin signaling and may improve memory, glucose metabolism, and cognitive functioning by this mechanism as well [30,35,89,112]. As a major cause of dementia and disability with relatively few therapeutic options on the horizon, IN DFO represents a promising yet relatively underdeveloped treatment for vascular dementia. Likewise, iron chelation has emerged as a neuroprotective strategy in a number of other neurological diseases including amyotrophic lateral sclerosis (ALS) [42] and PSP [71].

## 9. Conclusions

The complex and often progressive nature of neurological disease has undoubtedly contributed to the repeated failure of potential therapeutics. It is increasingly recognized that “silver bullets”—therapies that operate on one target within a cascade—are unlikely to be of major benefit when multiple mechanisms contribute to pathogenesis, as is the case in neurodegenerative and neurovascular disease. In the absence of measures for complete prevention or regeneration of lost brain function, so-called “dirty drugs” that operate on multiple events in these pathogenic cascades may be the only realistic future for meaningful benefit in these diseases, such as Alzheimer’s disease [88]. Herein, the evidence has been presented for IN DFO as one such therapy. Brain iron chelation may act as a general disease-nonspecific mechanism that engages multiple pathways, including downstream effectors of HIF-1α and insulin signaling, to intersect with and possibly slow the progression of multiple human neurological diseases including AD, PD, ischemic stroke, and ICH. Citing preclinical and early clinical work across decades, multiple research groups, alternative delivery mechanisms, and other iron chelators, we have argued that these putative mechanisms represent significant promise for translational efficacy. Intranasal delivery is poised to transform and mitigate the challenge of delivering pharmaceuticals to the CNS including DFO. By exerting its effects on multiple pathways including neurodegeneration, neuroinflammation, neurovascular dysfunction, and metabolic syndrome, IN DFO is positioned to have widespread benefit across neurodegenerative and neurovascular disease.
